# Baseline body mass index and early functional recovery after first recorded advanced therapy in rheumatoid arthritis: a real-world cohort study

**DOI:** 10.1007/s00296-026-06158-5

**Published:** 2026-05-28

**Authors:** Omer Denizhan Tatar, Levent Kılıç, Umut Kalyoncu, Ali Ihsan Ertenli

**Affiliations:** 1 Department of Internal Medicine, Hacettepe University Faculty of Medicine, Ankara, Turkey; 2 Department of Endocrinology and Metabolism, Necmettin Erbakan University Faculty of Medicine, Konya, Turkey; 3 Department of Rheumatology, Hacettepe University Faculty of Medicine, Ankara, Turkey

**Keywords:** Rheumatoid arthritis, Body mass index, Obesity, Antirheumatic agents, Treatment outcome, Disability evaluation

## Abstract

Obesity is common in rheumatoid arthritis (RA), but its prognostic value at first recorded advanced-therapy initiation is incompletely defined. We examined whether baseline body mass index (BMI) was associated with early DAS28-ESR remission and Health Assessment Questionnaire Disability Index (HAQ-DI)-based functional recovery after first recorded advanced therapy. Adults with clinician-diagnosed RA initiating first recorded advanced therapy between 2013 and 2020 were studied retrospectively. BMI was analyzed continuously and categorically. Outcomes were assessed at the eligible visit closest to 9 months within 6–12 months. Complete-case multivariable logistic regression was supplemented by robust Poisson and sensitivity analyses. Of 581 screened patients, 574 had baseline BMI data, including 235 (40.9%) with obesity. DAS28-ESR remission was evaluable in 258 patients and occurred in 125 (48.4%); remission was less frequent with obesity than with BMI < 25.0 or 25.0-29.9 kg/m² (38.7% vs. 55.8% and 54.7%). In the primary adjusted remission model (*n* = 208), BMI was not conventionally significant (OR 1.048 per 1 kg/m², 95% CI 0.999-1.100; *P* = 0.056). HAQ-DI response was evaluable in 228 patients; 226 entered the primary adjusted model. Higher BMI was associated with poor HAQ-DI response (OR 1.056, 95% CI 1.006–1.110; *P* = 0.029), attenuating after hypertension/diabetes adjustment. Higher BMI showed its most consistent adjusted association with less complete early HAQ-DI-based functional recovery, whereas the DAS28-ESR remission signal was weaker and model-sensitive. Findings are associative and prognostic rather than causal.

**Supplementary Information:**

The online version contains supplementary material available at 10.1007/s00296-026-06158-5.

## Introduction

Contemporary rheumatoid arthritis (RA) management follows treat-to-target principles, with remission preferred and low disease activity accepted when remission is not achievable [1]. Yet responses to biologic and targeted synthetic disease-modifying antirheumatic drugs remain heterogeneous in routine care, making accessible baseline markers relevant to early follow-up and shared decision-making [2–10].

This heterogeneity is intertwined with the cardiometabolic burden of RA. Hypertension, diabetes, dyslipidemia, obesity, and metabolic syndrome are frequent and may interact with systemic inflammation, cardiovascular risk, and long-term outcomes rather than simply coexist with RA [11–15]. Recent translational literature further supports interpreting RA within an integrated immunometabolic and cardiovascular-risk framework when evaluating treatment trajectories and patient-centered outcomes [16].

Obesity is a visible component of this inflammatory-metabolic interface. Although BMI does not measure adipose distribution or body composition, higher body size may reflect altered adipokine signaling, insulin resistance, low-grade inflammation, mechanical loading, pain amplification, and deconditioning, all of which may influence inflammatory indices and functional recovery [12, 13, 15, 17]. Prior cohorts and meta-analyses have linked overweight or obesity to lower odds of remission or favorable response, although findings vary across cohorts and mechanisms of action [2–10].

Recent Rheumatology International data highlighted overweight and obesity in real-world RA patients receiving biologic or targeted synthetic disease-modifying antirheumatic drugs [9]. We extend this journal-specific evidence by focusing on first recorded advanced-therapy initiation and by assessing early HAQ-DI-based functional recovery alongside DAS28-ESR remission.

Uncertainty remains because prior studies often emphasized inflammatory outcomes, pooled treatment lines, or focused on early RA rather than advanced-therapy initiation in routine-care cohorts [2–10]. Functional recovery also deserves attention because disability reflects inflammation, adiposity, pain, comorbidity, conditioning, and accumulated damage [18–22].

The aim of this study was to determine whether baseline BMI was associated with early DAS28-ESR remission and HAQ-DI-based functional recovery after first recorded advanced-therapy initiation in a tertiary real-world RA cohort. We also explored whether obesity with hypertension and/or diabetes identified a less favorable crude outcome profile. We hypothesized that higher BMI would be associated with lower remission probability and less complete functional improvement, while interpreting all analyses as observational and non-causal.

## Methods

### Study design and setting

This retrospective single-center cohort study was conducted in the tertiary rheumatology referral unit of a university hospital and was reported in accordance with the STROBE statement for observational studies [23]. A completed STROBE checklist is supplied as a supplementary file.

The center functions as a tertiary referral clinic and biologic registry site, so the cohort may include patients with more established disease, higher comorbidity burden, or more complex treatment histories than community-based RA cohorts. Treatment and follow-up decisions reflected physician discretion in routine care rather than a protocol-mandated algorithm, introducing possible referral and treatment-selection bias.

### Study population

Adults with clinician-diagnosed RA who initiated a first recorded advanced therapy between January 2013 and December 2020 were screened. Advanced therapy was defined as initiation of either a biologic disease-modifying antirheumatic drug (bDMARD) or a targeted synthetic disease-modifying antirheumatic drug (tsDMARD) [1]. Cohort entry was based on rheumatologist diagnosis during routine care; available records were reviewed for consistency with contemporary RA classification criteria when sufficient detail was available [24].

First recorded advanced therapy was defined as the first bDMARD or tsDMARD initiation captured in the institutional biologic registry. Because the retrospective registry could not fully exclude advanced-therapy exposure before registry capture for every patient, the term first recorded advanced therapy is used throughout the manuscript. Potential misclassification of true treatment line was considered an important limitation.

### Data source and baseline assessment

Clinical data were obtained from the institutional biologic registry of the participating tertiary center and cross-checked against electronic medical records when treatment dates, comorbidity status, or outcome measurements required clarification. Baseline was anchored to first recorded advanced-therapy initiation. BMI was calculated from routine-care height and weight at treatment initiation and interpreted as a pragmatic exposure rather than protocol-mandated anthropometry. Obesity was defined as BMI ≥ 30.0 kg/m², and BMI was analyzed continuously and as < 25.0, 25.0-29.9, and ≥ 30.0 kg/m² [17].

### Outcome definitions

The clinical outcome was failure to achieve DAS28-ESR remission during follow-up. Remission was defined as DAS28-ESR ≤ 2.6 and low disease activity as DAS28-ESR ≤ 3.2, consistent with conventional DAS28-ESR thresholds [25]. Follow-up disease activity was assessed at the eligible post-index visit closest to 270 days within a prespecified 182-365-day window after first recorded advanced-therapy initiation.

The functional outcome was poor HAQ-DI response, defined a priori as improvement < 0.22 over the same 182-365-day interval. HAQ-DI is a validated patient-reported disability measure in RA, and a change of approximately 0.22 is commonly used to represent clinically meaningful improvement [18, 19, 26, 27].

During data cleaning, follow-up HAQ-DI values outside the instrument scale range were considered implausible and recoded as missing. One follow-up HAQ-DI value > 3 met this criterion and was excluded from paired functional analyses.

### Covariates

Covariates were selected a priori on clinical grounds and with reference to prior RA outcomes literature [28]. The primary remission model included age, sex, registry-recorded RA duration at treatment initiation, baseline DAS28-ESR, hypertension, and diabetes mellitus. The primary functional model included age, sex, registry-recorded RA duration, baseline HAQ-DI, and baseline DAS28-ESR; hypertension and diabetes mellitus were added in a prespecified extended functional model to evaluate whether the BMI estimate was attenuated by cardiometabolic burden.

A directly recorded RA-duration variable was available, whereas diagnosis-date information was incomplete; therefore, registry-recorded duration was retained as the pragmatic disease-duration covariate. Smoking, seropositivity, methotrexate, glucocorticoid exposure, and coded first-agent treatment class were examined in sensitivity analyses. Treatment class was not forced into primary models because coding was incomplete; TNF inhibitor versus other coded first-agent class was used only as an exploratory robustness check, as granular non-TNF subclass coding was sparse.

Smoking and glucocorticoid exposure were also reparameterized more granularly, and reduced baseline glomerular filtration rate (< 90 mL/min/1.73 m²) was explored because renal data were more complete than lipid measurements. Lipid variables were too incomplete for stable multivariable adjustment. Adjusted estimates should therefore be interpreted as complete-case associative models, not fully adjusted causal estimates.

### Statistical analysis

Continuous variables were evaluated with Shapiro-Wilk tests and visual inspection of histograms and Q-Q plots. In accordance with the revision request, continuous descriptors are reported as median (minimum-maximum), and categorical variables as counts and percentages. Between-group comparisons used Kruskal-Wallis, chi-square, or Fisher exact tests, as appropriate. Interpretation emphasized estimates, confidence intervals, denominators, missingness, and model consistency rather than P values alone.

BMI was modeled primarily as a continuous exposure and re-examined as binary obesity and categorical BMI. Multivariable logistic regression was used for primary clinical and functional outcomes. Because both outcomes were common within the 6-12-month window, robust Poisson regression with sandwich standard errors was used as a scale sensitivity analysis. Additional models sequentially incorporated smoking, seropositivity, methotrexate, glucocorticoids, reduced baseline GFR, and exploratory treatment-class coding.

Primary adjusted estimates used complete-case analyses because outcome availability and several secondary covariates were incomplete and no defensible imputation model had been prespecified. Post hoc multiple imputation was not performed because outcome capture, treatment-class coding, and selected registry variables could not be confidently assumed missing at random. Characteristics of adjusted-model cohorts were compared with the broader baseline BMI cohort in the supplement.

Because baseline HAQ-DI and DAS28-ESR were entered together in the functional model, multicollinearity was checked using variance inflation factors. Quadratic BMI terms assessed departure from logit-linearity. Logistic models are reported as odds ratios (ORs) with 95% confidence intervals (CIs), and robust Poisson models as risk ratios (RRs). Analyses were rechecked against the original SPSS dataset and finalized with IBM SPSS Statistics version 25.0-compatible definitions. Two-sided P values < 0.05 were considered statistically significant, but borderline values were interpreted cautiously.

### Ethics

The study protocol was approved by the institutional non-interventional clinical research ethics committee; full approval details, including the committee name, project number, meeting number, decision number, and approval date, are provided in the title page/declarations. The requirement for written informed consent was waived by the approving committee because of the retrospective design and the use of routinely collected clinical data. The study was conducted in accordance with applicable institutional and national ethical standards and with the principles of the World Medical Association Declaration of Helsinki applicable at the time of ethics approval. During manuscript revision, the authors also considered the October 2024 revision of the Declaration of Helsinki and confirm that the study reporting and ethical safeguards remain consistent with applicable ethical principles.

## Results

### Cohort reconstruction and analytic denominators

A total of 581 patients with RA initiating first recorded advanced therapy were screened; 574 had baseline BMI and formed the exposure cohort. Eligible 6-12-month DAS28-ESR data were available for 258 patients, paired baseline/follow-up DAS28-ESR for 225, and paired HAQ-DI for 228 after exclusion of one implausible follow-up value. The primary remission model included 208 complete cases, mainly limited by missing baseline DAS28-ESR (*n* = 33) and diabetes status (*n* = 26), with overlap. The primary functional model included 226 patients; the extended functional model decreased to 208 after adding diabetes. Adjusted-model cohorts were broadly similar to the baseline BMI cohort but had somewhat longer recorded RA duration (Online Resource 1).

**Fig. 1:**
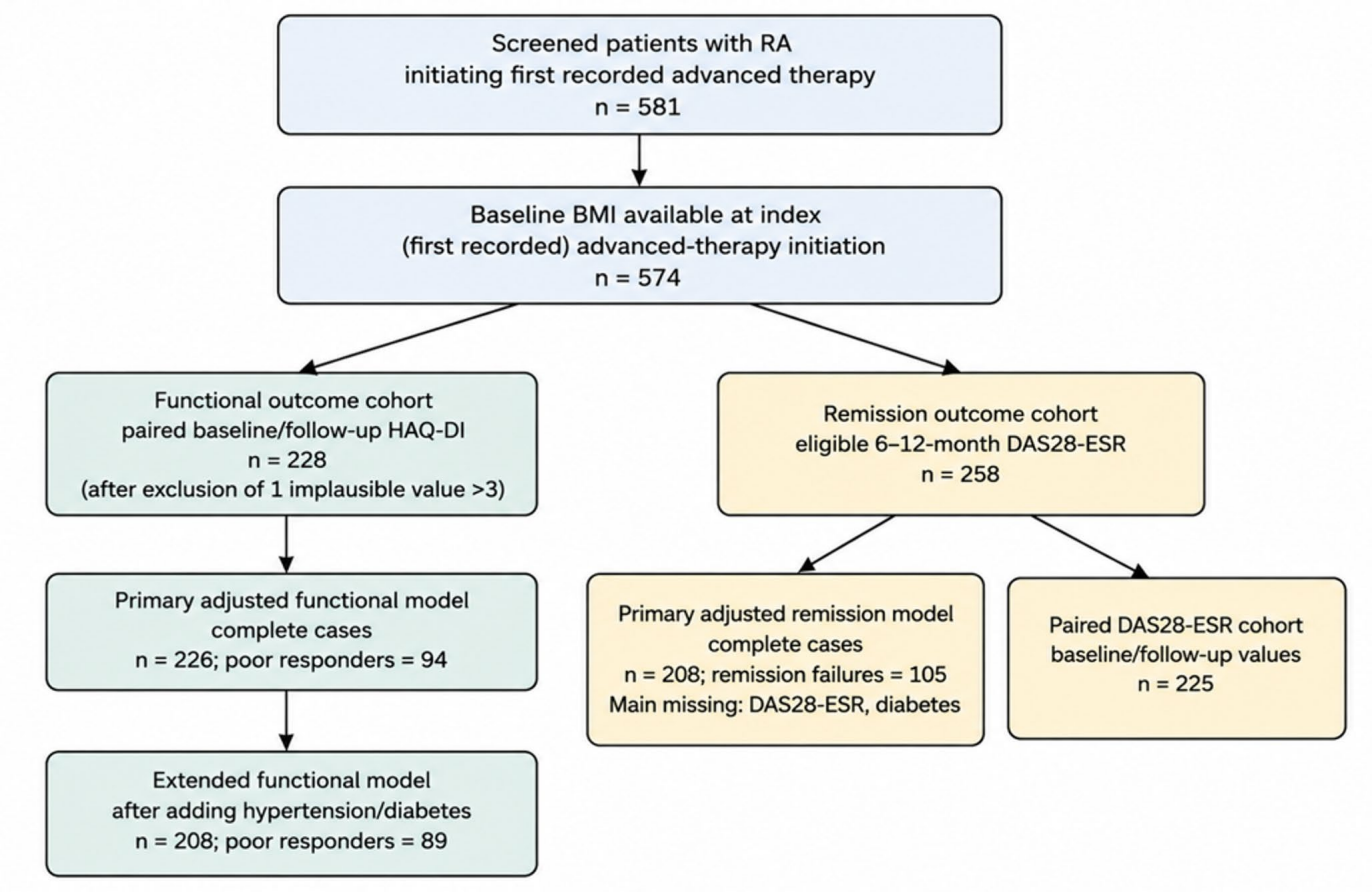
Flow of patients through the remission and functional analytic cohorts. DAS28-ESR, Disease Activity Score in 28 joints using erythrocyte sedimentation rate; HAQ-DI, Health Assessment Questionnaire Disability Index; RA, rheumatoid arthritis

The cohort flow is shown in Fig. 1.

### Baseline characteristics

The median age at first recorded advanced-therapy initiation was 50 years (range, 16–79), and 434/574 patients (75.6%) were women. Obesity was present in 235 patients (40.9%). Patients with obesity were older, more often female, and more frequently had hypertension or diabetes, whereas baseline DAS28-ESR was similar and baseline HAQ-DI modestly higher. Smoking, methotrexate, and glucocorticoid exposure were complete; serostatus was available for 553 patients; and first-agent treatment class for 357. Among coded starts, 242/357 (67.8%) were TNF inhibitors and 113/357 (31.7%) JAK inhibitors, with other non-TNF classes sparse. Baseline characteristics appear in Table 1 and Online Resource 1.

**Table 1:** Baseline characteristics according to BMI category at first recorded advanced-therapy initiation

Variable	Overall	<25.0	25.0-29.9	≥30.0	*P* value
Age at first recorded advanced therapy, years	50 (16-79)	40 (16-79)	52 (17-79)	53 (16-79)	<0.001
Female sex	434/574 (75.6%)	132/176 (75.0%)	110/163 (67.5%)	192/235 (81.7%)	0.005
Registry-recorded RA disease duration, years	3 (0-8)	3 (0-8)	3 (0-8)	3 (0-8)	0.597
Hypertension	164/574 (28.6%)	12/176 (6.8%)	52/163 (31.9%)	100/235 (42.6%)	<0.001
Diabetes mellitus	72/515 (14.0%)	7/151 (4.6%)	24/144 (16.7%)	41/220 (18.6%)	<0.001
Baseline DAS28-ESR	4.73 (1.47-8.08)	4.65 (1.47-7.55)	4.65 (1.47-7.78)	4.94 (1.47-8.08)	0.060
Baseline HAQ-DI	0.9 (0-2.95)	0.75 (0-2.9)	1 (0-2.9)	1 (0-2.95)	0.011

Continuous variables are reported as median (minimum-maximum), and categorical variables are reported as n/N (%). Between-group comparisons were evaluated using the Kruskal-Wallis test for continuous variables and chi-square or Fisher exact tests for categorical variables, as appropriate. P values are descriptive and were not adjusted for multiple testing. BMI categories are defined as <25.0, 25.0-29.9, and ≥30.0 kg/m². Diabetes mellitus denominators vary because baseline diabetes status was not available for all patients

### Clinical and functional outcomes across BMI categories

Among 258 patients with eligible 6-12-month DAS28-ESR data, 125 (48.4%) achieved remission. Remission occurred in 55.8%, 54.7%, and 38.7% of patients with BMI < 25.0, 25.0-29.9, and ≥ 30.0 kg/m², respectively (*P* = 0.032). Low disease activity showed a similar descriptive pattern (74.0%, 69.3%, and 57.5%; *P* = 0.051).

In the paired DAS28-ESR cohort, median DAS28-ESR decreased from 4.66 (range, 1.47–7.60) to 2.58 (range, 0.49–7.44). Follow-up DAS28-ESR was highest with obesity [2.92 (0.77–7.44)] and lower with BMI < 25.0 [2.41 (0.49–5.48)] or 25.0–29.9 kg/m² [2.33 (0.63–5.72); *P* = 0.009].

In the paired HAQ-DI cohort, median follow-up HAQ-DI was 0.35 (range, 0-2.20) and increased across BMI categories, reaching 0.50 (range, 0-2.05) in the obesity group (*P* < 0.001). Poor functional response occurred in 94/228 patients (41.2%) and was numerically more frequent across increasing BMI strata (34.2%, 42.6%, and 46.0%; *P* = 0.311). Median HAQ-DI improvement declined across BMI strata [0.40, 0.38, and 0.25; *P* = 0.107]. Outcomes are summarized in Table 2.

**Table 2:** Early clinical and functional outcomes according to baseline BMI category

Variable	Overall	<25.0	25.0-29.9	≥30.0	*P* value
DAS28-ESR remission at 6-12 months	125/258 (48.4%)	43/77 (55.8%)	41/75 (54.7%)	41/106 (38.7%)	0.032
DAS28-ESR low disease activity at 6-12 months	170/258 (65.9%)	57/77 (74.0%)	52/75 (69.3%)	61/106 (57.5%)	0.051
Baseline DAS28-ESR (paired cohort)	4.66 (1.47-7.6)	4.55 (1.6-6.93)	4.64 (1.47-7.6)	4.84 (1.47-7.32)	0.132
Follow-up DAS28-ESR (paired cohort)	2.58 (0.49-7.44)	2.41 (0.49-5.48)	2.33 (0.63-5.72)	2.92 (0.77-7.44)	0.009
Poor functional response (HAQ-DI improvement <0.22)	94/228 (41.2%)	25/73 (34.2%)	29/68 (42.6%)	40/87 (46.0%)	0.311
Follow-up HAQ-DI (paired cohort)	0.35 (0-2.2)	0.2 (0-1.3)	0.35 (0-2.2)	0.5 (0-2.05)	<0.001
HAQ-DI improvement (paired cohort)	0.35 (-1.15-1.9)	0.4 (-0.25-1.9)	0.38 (-0.85-1.85)	0.25 (-1.15-1.9)	0.107

Continuous variables are reported as median (minimum-maximum), and categorical variables are reported as n/N (%). Remission and low-disease-activity denominators are based on *n* = 258; paired DAS28-ESR analyses are based on *n* = 225; poor functional response and paired HAQ-DI analyses are based on *n* = 228. Between-group P values are unadjusted descriptive comparisons and do not replace the multivariable models. Remission and low disease activity were assessed in patients with an eligible 182-365-day DAS28-ESR value. Paired HAQ-DI analyses exclude one implausible follow-up HAQ-DI value outside the instrument range

### Exploratory cardiometabolic phenotype

In exploratory crude analyses, remission failure occurred in 43.0% of non-obese patients without hypertension/diabetes, 60.8% of patients with obesity alone, and 61.2% of patients with obesity plus hypertension and/or diabetes (*P* = 0.039). Poor functional response showed a similar but non-significant pattern (36.5%, 42.9%, and 52.4%; *P* = 0.214). These unadjusted, modest-size subgroup comparisons were considered descriptive and hypothesis-generating only (Tables 3 and 4).

**Table 3:** Logistic regression for failure to achieve DAS28-ESR remission at 6-12 months (primary complete-case model; *n* = 208)

Variable	Univariable OR (95% CI)	Univariable *P*	Multivariable OR (95% CI)	Multivariable *P*
Baseline BMI, per 1 kg/m²	1.051 (1.013-1.092)	0.009	1.048 (0.999-1.100)	0.056
Age, per year	1.021 (1.002-1.040)	0.033	1.024 (0.998-1.051)	0.076
Female sex	2.739 (1.536-4.885)	<0.001	2.867 (1.364-6.027)	0.005
Registry-recorded RA disease duration, per year	0.907 (0.803-1.025)	0.117	0.955 (0.821-1.111)	0.547
Baseline DAS28-ESR, per 1 point	1.422 (1.140-1.775)	0.002	1.364 (1.061-1.753)	0.015
Hypertension	1.333 (0.768-2.316)	0.307	0.894 (0.427-1.873)	0.767
Diabetes mellitus	1.059 (0.511-2.195)	0.876	0.689 (0.270-1.756)	0.435

The outcome was failure to achieve DAS28-ESR remission. Effect estimates are odds ratios with 95% confidence intervals. The multivariable model included baseline BMI, age, sex, registry-recorded RA duration, baseline DAS28-ESR, hypertension, and diabetes mellitus

**Table 4:** Logistic regression for poor functional response (HAQ-DI improvement <0.22) at 6-12 months (primary complete-case model; *n* = 226; extended model *n* = 208)

Variable	Univariable OR (95% CI)	Univariable *P*	Multivariable OR (95% CI)	Multivariable *P*
Baseline BMI, per 1 kg/m²	1.046 (1.006-1.087)	0.024	1.056 (1.006-1.110)	0.029
Age, per year	1.023 (1.002-1.045)	0.034	1.031 (1.004-1.059)	0.024
Female sex	1.430 (0.768-2.664)	0.259	3.003 (1.351-6.677)	0.007
Registry-recorded RA disease duration, per year	0.945 (0.826-1.082)	0.413	0.988 (0.841-1.161)	0.887
Baseline HAQ-DI, per 1 point	0.167 (0.092-0.301)	<0.001	0.132 (0.063-0.276)	<0.001
Baseline DAS28-ESR, per 1 point	0.576 (0.452-0.734)	<0.001	0.695 (0.515-0.938)	0.017

The outcome was poor functional response, defined as HAQ-DI improvement <0.22. Effect estimates are odds ratios with 95% confidence intervals. The primary multivariable model included baseline BMI, age, sex, registry-recorded RA duration, baseline HAQ-DI, and baseline DAS28-ESR. In the prespecified extended functional model additionally adjusted for hypertension and diabetes mellitus, the BMI estimate was attenuated (adjusted OR 1.043, 95% CI 0.990-1.099; *P* = 0.115; *n* = 208)

### Regression analyses

In univariable logistic regression, each 1 kg/m² higher BMI was associated with remission failure (OR 1.051, 95% CI 1.013–1.092; *P* = 0.009). In the prespecified multivariable model adjusted for age, sex, recorded RA duration, baseline DAS28-ESR, hypertension, and diabetes, the estimate remained similar but did not reach conventional significance (adjusted OR 1.048, 95% CI 0.999-1.100; *P* = 0.056; *n* = 208; 105 events). Female sex and higher baseline DAS28-ESR were independently associated with remission failure.

Binary-obesity and BMI-category models remained directionally similar but imprecise. Sequential adjustment for smoking, methotrexate, or glucocorticoid exposure yielded similar BMI estimates; seropositivity attenuated the estimate, and reduced baseline GFR strengthened it. A fuller sensitivity model including smoking, seropositivity, methotrexate, glucocorticoids, and GFR returned the BMI term to statistical uncertainty (adjusted OR 1.049, 95% CI 0.996–1.104; *P* = 0.070), supporting a model-sensitive remission signal.

The exploratory treatment-class model was much smaller (*n* = 123) and imprecise (adjusted OR 1.023, 95% CI 0.960–1.089; *P* = 0.485), so it was interpreted only as a limited robustness check. Robust Poisson analysis yielded an adjusted RR of 1.019 per 1 kg/m² higher BMI (95% CI 1.000-1.038; *P* = 0.045). A quadratic BMI term did not improve fit (*P* = 0.764).

Baseline BMI was more consistently associated with poor functional response. Each 1 kg/m² higher BMI was associated with poor HAQ-DI response in univariable analysis (OR 1.046, 95% CI 1.006–1.087; *P* = 0.024) and in the primary adjusted model (adjusted OR 1.056, 95% CI 1.006–1.110; *P* = 0.029; *n* = 226; 94 events). Older age and female sex were associated with poorer functional recovery. Higher baseline HAQ-DI and DAS28-ESR were inversely associated with poor response, likely reflecting greater measurable room for improvement; VIFs did not indicate problematic multicollinearity.

After additional adjustment for hypertension and diabetes, the BMI estimate attenuated (adjusted OR 1.043, 95% CI 0.990–1.099; *P* = 0.115; *n* = 208). Because these conditions are closely linked to adiposity, this was interpreted as a cardiometabolic-burden sensitivity model rather than the preferred total-effect model. Binary-obesity and BMI-category models were directionally consistent but imprecise.

Sensitivity analyses adding smoking, seropositivity, methotrexate, glucocorticoids, smoking-status coding, glucocorticoid-dose coding, or reduced baseline GFR preserved the functional BMI estimate. A fuller model including smoking, seropositivity, methotrexate, glucocorticoids, and GFR remained significant (adjusted OR 1.056, 95% CI 1.004–1.112; *P* = 0.036; *n* = 212). The exploratory treatment-class model was smaller and imprecise. Robust Poisson analysis yielded an adjusted RR of 1.028 (95% CI 1.007–1.049; *P* = 0.007), and a quadratic BMI term did not improve fit (Table 5).

**Table 5:** Analytic denominators and main sources of complete-case restriction

Domain	Available cohort	Primary adjusted model	Main reason for reduction	Interpretive implication
Baseline BMI cohort	574	Not applicable	7/581 screened patients lacked baseline BMI	Baseline analytic cohort for exposure analyses
DAS28-ESR remission analysis	258 eligible 6-12-month DAS28-ESR assessments	208 complete cases	Missing baseline DAS28-ESR (*n* = 33) and diabetes-status entries (*n* = 26), with 9 patients overlapping across both missingness domains	Primary remission model is complete-case and should be interpreted cautiously
Paired DAS28-ESR descriptive cohort	225 paired baseline and follow-up DAS28-ESR values	Not applicable	Incomplete baseline or follow-up DAS28-ESR	Descriptive change analyses use paired data only
HAQ-DI functional analysis	228 paired baseline and follow-up HAQ-DI observations	226 complete cases	One implausible follow-up HAQ-DI >3 recoded as missing; 2 patients lacked complete model covariates	Primary functional model preserves nearly all eligible paired HAQ-DI patients
Extended functional model	228 paired baseline and follow-up HAQ-DI observations	208 complete cases	Addition of diabetes status reduced complete-case sample	Extended model is a cardiometabolic-burden sensitivity model

This table was added to make the analytic denominators and complete-case restrictions more transparent in response to reviewer concerns about data loss and included-versus-excluded comparisons

## Discussion

In this tertiary real-world RA cohort, higher baseline BMI was associated with a less favorable early response pattern after first recorded advanced-therapy initiation. Patients with obesity had lower crude remission rates, higher follow-up DAS28-ESR, and higher follow-up disability. After adjustment, however, the remission estimate was borderline and model-sensitive, whereas the HAQ-DI association was more consistent. These findings support a modest prognostic signal, strongest for functional recovery, rather than a causal or mechanism-specific effect.

This separation matters clinically. BMI is readily available at treatment escalation, but its prognostic meaning is embedded within broader metabolic and functional context. Additional adjustment for smoking, seropositivity, methotrexate, glucocorticoids, and reduced GFR had little effect on the functional estimate, whereas remission was more model-sensitive. BMI may therefore contextualize early functional recovery without serving as an isolated biological determinant of advanced-therapy response.

Our findings align with prior evidence linking obesity to less favorable RA outcomes. Reviews, early RA cohorts, and registry analyses have reported lower remission rates or less favorable response among patients with overweight or obesity [2–6, 8–10], although some mechanism-specific datasets, including tocilizumab registry data, suggest preserved effectiveness across obesity strata [7]. Recent Rheumatology International registry data also linked overweight/obesity with RA outcomes [9]. Our study adds a narrower decision-point perspective and suggests that the most reproducible adjusted signal may involve functional recovery rather than DAS28-ESR remission.

The HAQ-DI finding deserves emphasis because disability reflects pain, conditioning, comorbidity, joint damage, and body size, not inflammation alone [18–21]. The inverse associations of baseline HAQ-DI and DAS28-ESR with poor functional response should not be interpreted as protective; they likely reflect greater room for measurable improvement and the structure of change-threshold outcomes. Regression-to-the-mean and measurement-structure effects cannot be excluded.

The exploratory obesity-plus-comorbidity grouping suggested a less favorable crude outcome profile among patients with obesity plus hypertension and/or diabetes. Because subgroup analyses were unadjusted and modest in size, they cannot establish effect modification or support definitive clinical conclusions. They should be viewed only as hypothesis-generating and as a rationale for prospective metabolic-phenotype studies [8, 12–14].

Several limitations should be considered. The retrospective single-center design limits generalizability and precludes causal inference. The tertiary referral setting may enrich for complex disease or comorbidity. Physician-directed treatment decisions introduce treatment-selection bias and confounding by indication.

Analytic sample size declined because 6-12-month outcome capture and selected covariates were incomplete. Primary models therefore used complete cases. Adjusted-model patients were broadly similar to the baseline BMI cohort for measured characteristics but had longer recorded RA duration; unmeasured missingness mechanisms, including missing-not-at-random processes, remain possible. Multiple imputation was not performed because outcome availability, treatment class, and registry covariates could not be modeled confidently under a missing-at-random assumption.

Treatment-class coding was incomplete, limiting adjustment for therapeutic mechanism and leaving residual confounding by treatment selection. The TNF-inhibitor-versus-other sensitivity model was smaller and imprecise, so it was not considered definitive. Potential unrecorded advanced-therapy exposure before registry capture may have misclassified true treatment line. BMI also cannot distinguish adiposity from lean mass, central adiposity, sarcopenic obesity, or rheumatoid cachexia [15, 17]. Radiographic damage, fibromyalgia or central pain amplification, physical activity, and detailed metabolic phenotyping were unavailable.

Other analytic limitations include one implausible follow-up HAQ-DI value treated as missing and use of a HAQ-DI change-threshold outcome adjusted for baseline HAQ-DI. The functional model should therefore be read as a clinically anchored prognostic model rather than an etiologic decomposition of disability change. Because outcomes were common, logistic ORs should not be read as risk ratios, although robust Poisson analyses were concordant.

Strengths include a routine tertiary-care cohort, a clinically relevant first recorded advanced-therapy initiation point, a prespecified follow-up window, and parallel assessment of clinical remission and patient-centered function. Model complexity was proportionate to event counts, and findings were examined through sensitivity models, alternative BMI parameterizations, robust Poisson scaling, linearity checks, and collinearity diagnostics.

In summary, higher baseline BMI was associated with a less favorable early response pattern after first recorded advanced therapy, with the most consistent adjusted signal for HAQ-DI-based functional recovery. The DAS28-ESR remission association was weaker, conventionally non-significant in the prespecified model, and more model-sensitive. BMI should be viewed as a pragmatic prognostic marker within a broader metabolic and functional profile, not a stand-alone causal determinant.

## Conclusions

Higher baseline BMI was associated with a modestly less favorable early treatment profile after first recorded advanced therapy in this real-world RA cohort. The most consistent adjusted finding involved HAQ-DI-based functional recovery, whereas the DAS28-ESR remission signal was weaker and model-sensitive. Obesity with hypertension and/or diabetes identified a less favorable unadjusted profile but remains hypothesis-generating. BMI and metabolic context may help interpret early RA treatment response without implying that BMI is a stand-alone causal determinant.

## Supplementary Information

Below is the link to the electronic supplementary material.

Supplementary Material 1

## Data Availability

The datasets generated and/or analyzed during the current study are not publicly available because they contain potentially identifiable patient-level clinical information and are subject to institutional and ethical restrictions. De-identified data may be made available from the corresponding author on reasonable request and subject to approval from the relevant institutional authorities.

## Declarations

### Related congress abstract publication

Preliminary findings from the same institutional RA/bDMARD cohort were previously presented as poster abstract PP-032 at the XXII. Ulusal Romatoloji Kongresi (XXII National Rheumatology Congress), 26–30 October 2022, Türkiye, and published in the congress abstract supplement. The abstract focused on obesity prevalence and disease activity among patients with RA receiving bDMARD therapy. The present manuscript represents a substantially expanded and distinct full-length analysis focusing on baseline BMI and early DAS28-ESR/HAQ-DI outcomes after first recorded advanced therapy. Tatar OD, Sandal Uzun G, Bilgin E, Yardımcı GK, Bölek EÇ, Farisoğulları B, Duran E, Ayan G, Özsoy Z, Ekici M, Ünaldı E, Kılıç L, Akdoğan A, Karadağ Ö, Apraş Bilgen Ş, Kiraz S, Kalyoncu U, Ertenli Aİ. bDMARD tedavisi kullanan romatoid artrit hastalarında obezite sıklığı ve hastalık aktivitesine etkisi. PP-032. Journal of Turkish Society for Rheumatology. 2022;14(Suppl 1):60–61. DOI: not available.

### Disclaimer

This manuscript has not been published previously as a full-length journal article, and no part of the submitted manuscript text has been copied from or published elsewhere, in whole or in part. The relationship of this work to the corresponding author’s 2022 specialty thesis and to the previously published congress abstract is disclosed above.
