# Supplementary material for: Baseline body mass index and early functional recovery after first recorded advanced therapy in rheumatoid arthritis: a real-world cohort study
Source: Rheumatol Int. 2026 May 28;46(6):117. doi: 10.1007/s00296-026-06158-5 (PMC13219159; doi:10.1007/s00296-026-06158-5)
Supplement: Supplementary file 1 — Supplementary Material 1 [file 296_2026_6158_MOESM1_ESM.docx]

**Online Resource 1**

Supplementary tables

Article title: Baseline Body Mass Index and Early Functional Recovery After First Recorded Advanced Therapy in Rheumatoid Arthritis: A Real-World Cohort Study

Journal: Rheumatology International

**Supplementary Table S1. Additional baseline covariates according to BMI category at first recorded advanced-therapy initiation.**

| **Variable** | **<25.0** | **25.0-29.9** | **≥30.0** | **P value** |
| --- | --- | --- | --- | --- |
| Ever smoking | 93/176 (52.8%) | 85/163 (52.1%) | 109/235 (46.4%) | 0.35 |
| RF and/or anti-CCP positivity | 111/166 (66.9%) | 113/157 (72.0%) | 149/230 (64.8%) | 0.327 |
| Concomitant methotrexate use | 105/176 (59.7%) | 89/163 (54.6%) | 143/235 (60.9%) | 0.439 |
| Baseline glucocorticoid use | 162/176 (92.0%) | 142/163 (87.1%) | 213/235 (90.6%) | 0.295 |
| Glucocorticoid dose >5 mg/day | 70/176 (39.8%) | 63/163 (38.7%) | 73/235 (31.1%) | 0.131 |
| TNF inhibitor among coded first-agent classes | 77/113 (68.1%) | 69/102 (67.6%) | 96/142 (67.6%) | 0.995 |

Note: Values are n/N (%). P values are descriptive and were obtained using chi-square tests with available-case denominators. Combined serostatus was available for 553 of 574 baseline-BMI patients. First-agent treatment class was coded for 357 patients; the stable treatment-class sensitivity term was TNF inhibitor versus other coded first-agent category because more granular non-TNF biologic/tsDMARD coding was sparse or incomplete in the source registry.

**Supplementary Table S2. Expanded sensitivity analyses and alternative covariate coding: adjusted association between baseline BMI and 6-12-month outcomes.**

| **Model specification** | **n** | **Events** | **Adjusted OR per 1 kg/m²** | **95% CI** | **P value** |
| --- | --- | --- | --- | --- | --- |
| Failure to achieve DAS28-ESR remission: Primary multivariable model (reference) | 208 | 105 | 1.048 | 0.999-1.100 | 0.056 |
| Failure to achieve DAS28-ESR remission: Smoking exposure | 208 | 105 | 1.048 | 0.999-1.100 | 0.055 |
| Failure to achieve DAS28-ESR remission: Smoking status (never/former/current) | 208 | 105 | 1.049 | 0.999-1.100 | 0.053 |
| Failure to achieve DAS28-ESR remission: Baseline seropositivity | 203 | 105 | 1.04 | 0.990-1.093 | 0.118 |
| Failure to achieve DAS28-ESR remission: Concomitant methotrexate use | 208 | 105 | 1.046 | 0.997-1.098 | 0.066 |
| Failure to achieve DAS28-ESR remission: Baseline glucocorticoid use | 208 | 105 | 1.05 | 1.000-1.102 | 0.049 |
| Failure to achieve DAS28-ESR remission: Glucocorticoid dose >5 mg/day | 208 | 105 | 1.048 | 0.999-1.100 | 0.055 |
| Failure to achieve DAS28-ESR remission: Reduced baseline GFR (<90 mL/min/1.73 m²) | 207 | 104 | 1.057 | 1.006-1.110 | 0.029 |
| Failure to achieve DAS28-ESR remission: Smoking + seropositivity + methotrexate + glucocorticoid + reduced GFR | 202 | 104 | 1.049 | 0.996-1.104 | 0.07 |
| Failure to achieve DAS28-ESR remission: Exploratory TNF inhibitor vs other coded first agent | 123 | 61 | 1.023 | 0.960-1.089 | 0.485 |
| Poor HAQ-DI response: Primary multivariable model (reference) | 226 | 94 | 1.056 | 1.006-1.110 | 0.029 |
| Poor HAQ-DI response: Primary model + hypertension and diabetes mellitus | 208 | 89 | 1.043 | 0.990-1.099 | 0.115 |
| Poor HAQ-DI response: Smoking exposure | 226 | 94 | 1.057 | 1.006-1.110 | 0.029 |
| Poor HAQ-DI response: Smoking status (never/former/current) | 226 | 94 | 1.055 | 1.004-1.109 | 0.034 |
| Poor HAQ-DI response: Baseline seropositivity | 221 | 92 | 1.055 | 1.003-1.110 | 0.037 |
| Poor HAQ-DI response: Concomitant methotrexate use | 226 | 94 | 1.056 | 1.006-1.110 | 0.029 |
| Poor HAQ-DI response: Baseline glucocorticoid use | 226 | 94 | 1.058 | 1.007-1.112 | 0.026 |
| Poor HAQ-DI response: Glucocorticoid dose >5 mg/day | 226 | 94 | 1.057 | 1.006-1.111 | 0.028 |
| Poor HAQ-DI response: Reduced baseline GFR (<90 mL/min/1.73 m²) | 217 | 91 | 1.055 | 1.004-1.109 | 0.034 |
| Poor HAQ-DI response: Smoking + seropositivity + methotrexate + glucocorticoid + reduced GFR | 212 | 89 | 1.056 | 1.004-1.112 | 0.036 |
| Poor HAQ-DI response: Exploratory TNF inhibitor vs other coded first agent | 133 | 58 | 1.03 | 0.970-1.093 | 0.333 |

Note: Primary remission models used age, sex, recorded RA duration, baseline DAS28-ESR, hypertension, and diabetes mellitus as the base covariate set. Primary functional models used age, sex, recorded RA duration, baseline HAQ-DI, and baseline DAS28-ESR; the prespecified extended functional model additionally included hypertension and diabetes mellitus. Reduced baseline GFR was coded as <90 mL/min/1.73 m². Treatment-class analyses were exploratory because first-agent coding was incomplete in the source registry. These sensitivity analyses were used to evaluate robustness and residual confounding structure rather than as a separate confirmatory inferential layer.

**Supplementary Table S3. Robust Poisson sensitivity analyses for the primary outcome models.**

| **Outcome** | **Model specification** | **n** | **Events** | **Adjusted RR per 1 kg/m²** | **95% CI** | **P value** |
| --- | --- | --- | --- | --- | --- | --- |
| Failure to achieve DAS28-ESR remission | Primary robust Poisson model | 208 | 105 | 1.019 | 1.000-1.038 | 0.045 |
| Poor functional response (HAQ-DI improvement <0.22) | Primary robust Poisson model | 226 | 94 | 1.028 | 1.007-1.049 | 0.007 |

Note: Because remission failure and poor functional response were common within the fixed 6-12-month assessment window, robust Poisson regression with sandwich standard errors was used as a scale sensitivity analysis. Effect estimates are reported as adjusted risk ratios (RRs) per 1 kg/m² higher baseline BMI using the same complete-case covariate sets as the corresponding primary logistic models.

**Supplementary Table S4. Comparison of the broader baseline BMI cohort with the adjusted remission and primary functional model cohorts.**

| **Variable** | **Baseline BMI cohort** | **Adjusted remission model cohort** | **Adjusted functional model cohort** |
| --- | --- | --- | --- |
| Age at first recorded advanced therapy, years | 50 (16-79) | 51 (18-79) | 50 (21-79) |
| Baseline BMI, kg/m² | 28.35 (15.62-58) | 27.9 (15.62-57.53) | 27.47 (15.62-57.53) |
| Registry-recorded RA disease duration, years | 3 (0-8) | 4 (0-8) | 4 (1-8) |
| Baseline DAS28-ESR | 4.73 (1.47-8.08) | 4.68 (1.47-7.6) | 4.66 (1.47-7.6) |
| Baseline HAQ-DI | 0.9 (0-2.95) | 0.9 (0-2.5) | 0.9 (0-2.5) |
| Female sex | 434/574 (75.6%) | 158/208 (76.0%) | 170/226 (75.2%) |
| Hypertension | 164/574 (28.6%) | 59/208 (28.4%) | 60/226 (26.5%) |
| Diabetes mellitus | 72/515 (14.0%) | 27/208 (13.0%) | 29/208 (13.9%) |
| Ever smoking | 287/574 (50.0%) | 111/208 (53.4%) | 121/226 (53.5%) |
| RF and/or anti-CCP positivity | 373/553 (67.5%) | 135/203 (66.5%) | 153/221 (69.2%) |
| Concomitant methotrexate use | 337/574 (58.7%) | 124/208 (59.6%) | 139/226 (61.5%) |
| Baseline glucocorticoid use | 517/574 (90.1%) | 186/208 (89.4%) | 205/226 (90.7%) |

Note: Continuous variables are reported as median (minimum-maximum), and categorical variables are reported as n/N (%). The adjusted remission model cohort refers to the 208 complete cases in the primary remission model. The adjusted functional model cohort refers to the 226 complete cases in the primary functional model. Diabetes mellitus denominators vary because baseline diabetes-status data were incomplete in the source registry.

**Supplementary Table S5. First-agent treatment-class coding completeness and granular distribution among coded first agents.**

| **Treatment-class coding item** | **n/N (%)** | **Comment** |
| --- | --- | --- |
| First-agent treatment class coded | 357/574 (62.2%) | Available for less than two thirds of the baseline BMI cohort |
| First-agent treatment class unavailable | 217/574 (37.8%) | Main reason treatment class was not forced into primary models |
| TNF inhibitor among coded first-agent classes | 242/357 (67.8%) | Stable exploratory treatment-class term used in sensitivity analyses |
| JAK inhibitor among coded first-agent classes | 113/357 (31.7%) | Dominant non-TNF coded first-agent category |
| Rituximab among coded first-agent classes | 1/357 (0.3%) | Sparse cell; not suitable for granular multivariable adjustment |
| IL-1 inhibitor among coded first-agent classes | 1/357 (0.3%) | Sparse cell; not suitable for granular multivariable adjustment |
| IL-6 inhibitor among coded first-agent classes | 0/357 (0.0%) | No coded initial starts in the available first-agent field |
| Abatacept among coded first-agent classes | 0/357 (0.0%) | No coded initial starts in the available first-agent field |
| Exploratory remission model with TNFi vs other coded first agent | n = 123; 61 events | BMI estimate was imprecise: adjusted OR 1.023, 95% CI 0.960-1.089; P = 0.485 |
| Exploratory functional model with TNFi vs other coded first agent | n = 133; 58 events | BMI estimate was imprecise: adjusted OR 1.030, 95% CI 0.970-1.093; P = 0.333 |

Note: This table was added to clarify why treatment class was not included in the primary models. Although the source registry supported a stable TNF inhibitor versus other coded first-agent sensitivity term, missingness and sparse non-TNF subclasses prevented definitive granular adjustment for IL-6 inhibitors, abatacept, rituximab, and JAK inhibitors.

**Supplementary Table S6. Missingness and analytic-denominator summary.**

| **Analytic step** | **n** | **Key missingness/data-quality issue** | **Manuscript handling** |
| --- | --- | --- | --- |
| Patients screened | 581 | Not all screened patients had baseline BMI | Baseline BMI cohort restricted to n = 574 |
| Baseline BMI cohort | 574 | 7 screened patients lacked baseline BMI | Main exposure cohort |
| Eligible remission outcome cohort | 258 | Outcome capture within 182-365 days unavailable for remaining patients | Primary remission outcome denominator |
| Primary adjusted remission model | 208 | Missing baseline DAS28-ESR (n = 33) and diabetes status (n = 26), with 9 patients overlapping | Complete-case model; selection discussed as limitation |
| Paired DAS28-ESR cohort | 225 | Baseline and follow-up DAS28-ESR pair required | Descriptive paired disease-activity analyses |
| Paired HAQ-DI cohort | 228 | One follow-up HAQ-DI value >3 was implausible and excluded | Functional outcome denominator |
| Primary adjusted functional model | 226 | Two patients lacked complete model covariates | Near-complete functional complete-case model |
| Extended functional model | 208 | Diabetes-status missingness after cardiometabolic extension | Sensitivity model, not preferred total-effect model |

Note: This table was added to make complete-case restriction more visible and to support the interpretation that the remission model is more vulnerable to outcome/covariate missingness than the primary functional model.

**Supplementary Table S7. Normality assessment for continuous descriptive variables.**

| **Variable** | **n** | **Median (Min-Max)** | **Shapiro-Wilk P** |
| --- | --- | --- | --- |
| Age at first recorded advanced therapy | 574 | 50 (16-79) | <0.001 |
| Registry-recorded RA disease duration | 574 | 3 (0-8) | <0.001 |
| Baseline DAS28-ESR | 484 | 4.73 (1.47-8.08) | 0.053 |
| Baseline HAQ-DI | 485 | 0.9 (0-2.95) | <0.001 |
| Follow-up DAS28-ESR | 225 | 2.58 (0.49-7.44) | <0.001 |
| Follow-up HAQ-DI | 228 | 0.35 (0-2.2) | <0.001 |
| HAQ-DI improvement | 228 | 0.35 (-1.15-1.9) | 0.002 |

Note: Shapiro-Wilk tests were used as distributional checks together with visual inspection of histograms and Q-Q plots. Because several descriptors were non-normally distributed and the editor specifically requested median (minimum-maximum) reporting, continuous variables were reformatted as median (minimum-maximum) in the revised descriptive tables.
